# Tetrachloridocuprates(II)—Synthesis and Electron Paramagnetic Resonance (EPR) Spectroscopy

**DOI:** 10.3390/ijms13021612

**Published:** 2012-02-02

**Authors:** Alette Winter, André Zabel, Peter Strauch

**Affiliations:** Institute of Chemistry, University of Potsdam, Karl-Liebknecht-Str. 24-25, D-14476 Potsdam/Golm, Germany; E-Mails: awinter@uni-potsdam.de (A.W.); azabel@uni-potsdam.de (A.Z.)

**Keywords:** tetrachloridocuprate(II), electron paramagnetic resonance, copper(II), ionic liquid

## Abstract

Ionic liquids (ILs) on the basis of metal containing anions and/or cations are of interest for a variety of technical applications e.g., synthesis of particles, magnetic or thermochromic materials. We present the synthesis and the results of electron paramagnetic resonance (EPR) spectroscopic analyses of a series of some new potential ionic liquids based on tetrachloridocuprates(II), [CuCl_4_]^2−^, with different sterically demanding cations: hexadecyltrimethylammonium **1**, tetradecyltrimethylammonium **2**, tetrabutylammonium **3** and benzyltriethylammonium **4**. The cations in the new compounds were used to achieve a reasonable separation of the paramagnetic Cu(II) ions for EPR spectroscopy. The EPR hyperfine structure was not resolved. This is due to the exchange broadening, resulting from still incomplete separation of the paramagnetic Cu(II) centers. Nevertheless, the principal values of the electron Zeemann tensor (*g_║_* and *g*_┴_) of the complexes could be determined. Even though the solid substances show slightly different colors, the UV/Vis spectra are nearly identical, indicating structural changes of the tetrachloridocuprate moieties between solid state and solution. The complexes have a promising potential e.g., as high temperature ionic liquids, as precursors for the formation of copper chloride particles or as catalytic paramagnetic ionic liquids.

## 1. Introduction

Ionic liquids (ILs) or ionic liquid crystals (ILCs) on the basis of metal containing anions and/or cations are of interest for a variety of technical applications e.g., as precursors for synthesis of (nano)particles, magnetic or thermochromic materials [1–3] or for usage as extraction agents [4] or catalysts [5–8]. Tetrahalidometalate complexes can combine many of these desired properties: Liquid-crystalline ionic liquids based on tetrahalidometalates (M = Co, Ni) have been reported first in 1996 by C.J. Bowlas, D.W. Bruce and K.R. Seddon [9]. In 2001 F. Neve *et al.* [10–13] showed that tetrahalidocuprate(II) complexes with alkylpyridinium cations including an alkyl chain length of *n* ≥ 12 are thermotropic liquid crystals. With shorter alkyl chains, they are ionic liquids. Additionally, alkylpyridinium tetrahalidocuprate ILs and ILCs have also been used as precursors for inorganic materials [2,14,15]. For the purpose of a deeper understanding of the thermotropic behavior of these *N*-alkylpyridinium salts, they were further investigated starting from the chain length of *n* = 9–18 [10,11]. R.D. Willett *et al.* already reported in 1974 one of the most striking examples of the copper(II) chloride complexes characterized by the phenomena of thermochromism which is interpreted in the same work as a phase transition leading to the change in the coordination geometry [16]. The compound forms bright green crystals at room temperature. When heated above 43 °C, the compound suddenly turns yellow and the color change is reversible. The compounds of our series do not show any thermochromic behavior in the investigated temperature range (up to 120 °C).

Metal containing ionic liquids provide also the interesting feature of being electrochromic and/or magnetic [17,18]. With their already known properties such as tunable acidity, polarity, amphiphilic character, coordinating ability, and miscibility with various compounds and properties due to the metal ion, these ILs could be the best candidates as catalysts for many chemical reactions as well as alternative solvents and morphology templates for inorganic materials at the same time. However, reports on tetrahalidometalate-based ILs/ILCs and their applications as well as their characterization are relatively rare [2,10–13,19–32]. This is somewhat surprising because metal containing ILs and ILCs may open the door towards a new valuable branch of IL research, due to its additional properties such as color, geometry, and magnetism, proffered by the metal ion composition.

**Figure 1:**
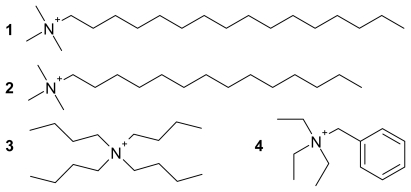
Cations of the CuCl_4_ salts **1**–**4**.

We present the syntheses and the results of electron paramagnetic resonance (EPR) spectroscopic analyses including temperature dependent measurements of some new tetrachloridocuprate(II) compounds with the following sterically demanding cations: hexadecyltrimethylammonium (C_16_H_33_Me_3_N^+^), tetradecyltrimethylammonium (C_14_H_29_Me_3_N^+^), tetrabutylammonium (Bu_4_N^+^) and benzyltriethylammonium (BzlEt_3_N^+^) ions (see Figure 1). They were used to achieve a reasonable separation of the paramagnetic Cu(II) ions for EPR spectroscopy.

## 2. Results and Discussion

EPR spectroscopy offers a good method for the investigation of Cu(II) compounds because of its high sensitivity towards diamagnetic systems and the accessibility to measure the samples in various physical states, *i.e.*, liquids and solids (powder, crystal and frozen solution); moreover the investigation of flexible systems like ILs on the basis of tetrachloridocuprate(II). For EPR investigations of pure ionic liquids or crystalline powder samples one problem arises. The paramagnetic interactions usually exceed the limits of the individual complex. Therefore, we use sterically rather demanding ammonium cations to separate the paramagnetic tetrachloridocuprate(II) moieties to achieve a minimum of magnetic interactions between the Cu(II) centers. Nevertheless, due to line broadening because of an incomplete separation of the paramagnetic centers, no hyperfine structure was observed (^63^Cu, ^65^Cu; nuclear spin *I* = 3/2 each). In addition, the four chlorido ligands lead to a further splitting of the signals (^35^Cl, ^37^Cl; nuclear spin *I* = 3/2 each) which is also not resolved and contributes to the line broadening in the EPR spectra. Finally, we succeeded in recording EPR spectra of our series which allowed us to determine the *g*-tensor parameters (*g_║_* and *g*_┴_).

**Figure 2:**
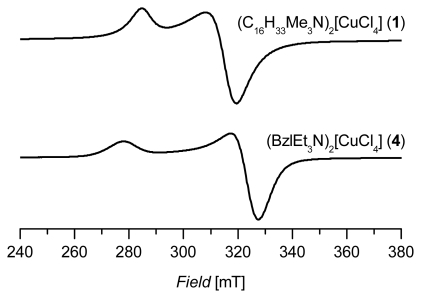
X-band powder electron paramagnetic resonance (EPR) spectra of **1** and **4** measured at 150 K.

Figure 2 shows two characteristic spectra of powdered samples of this series (C_16_H_33_Me_3_N)_2_[CuCl_4_] (**1**) and (BzlEt_3_N)_2_[CuCl_4_] (**4**). The spectra are all of axial symmetry and *g*_║_ as well as *g*_┴_ can be determined (see Table 1). In Table 1 the averaged *g*-values *g*_av_ calculated from the following simple expression for axial symmetry are also listed:

(1) $$g_{\text{av}}=\frac{g_{\left|\right|}+2\cdot g_{⊥}}{3}$$

The *g*_av_-value corresponds to the isotropic *g*_iso_-value of liquid systems (e.g., solutions or ionic liquids). The table also contains an example of a diamagnetically diluted ionic liquid bis(n-butylpyridinium)tetrachloridocuprate/zincate(II), (BuPy)_2_[Cu/ZnCl_4_]. In this spectrum the parallel part of the copper hyperfine structure is reasonably resolved [33]. The variation of the parameters reflects the degree of structural flexibility of the tetrachloridocuprate(II) moiety. With a series of known X-ray structures combined with EPR parameters of tetrachloridocuprates(II), it was possible to classify the degree of distortion between square planar and tetrahedral geometries, as it was recently shown for tetrabromidocuprates(II) [34].

**Figure 3:**
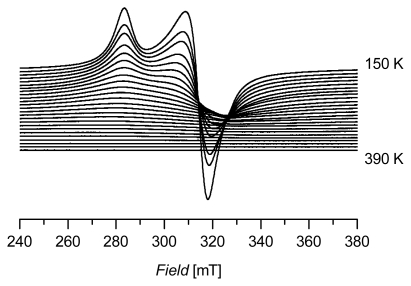
X-band EPR spectra of **2** (measured in a temperature range from 150 to 390 K in 10 K steps).

A sample of (C_14_H_29_Me_3_N)_2_[CuCl_4_] (**2**), was investigated by EPR at various temperatures. Figure 3 summarizes the results of the X-band EPR measurements of a powdered sample of **2**, measured in a temperature range from 150 to 390 K in 10 K steps. The spectrum at 150 K is comparable to the spectra shown in Figure 2. Interestingly, the signal continuously decreases with rising temperatures and disappears fully above ~350 K. The significant decrease in intensity of the spectra might be attributed to saturation effects depending on the energy gap between the ground state and the first excited state in the [CuCl_4_]^2−^ unit. This effect is completely reversible in the investigated temperature range, indicating that the compound is also stable at higher temperatures without any signs of decomposition (at least at 390 K).

## 3. Experimental Section

### 3.1. Chemicals

The following chemicals were used without further purification. Copper(II) chloride (98%), copper(II) chloride dihydrate (99%), hydrochloric acid (37%), ethanol anhydrous (99%), potassium bromide (Uvasol, for IR spectroscopy), hexadecyltrimethylammonium chloride (>95%), tetrabutylammonium chloride (95%), benzyltriethylammonium chloride (>98%), tetradecyltrimethylammonium chloride (>98%), 2-propanol (99.5%), diisopropyl ether (100%).

### 3.2. Preparation

In general, tetrachloridocuprate(II) complexes can be achieved by different procedures [35–38], three of them are mentioned here. (1) At first the internal [CuCl_4_]^2−^ coordination sphere is prepared by dissolving copper(II) chloride dihydrate in hydrochloric acid (37% wt). This is followed by addition of the counter ion as chloride salt dissolved in 2-propanol. After 30 minutes heating under reflux the solvent is evaporated until the complex precipitates [36]; (2) In a second synthetic protocol the internal coordination sphere is obtained in situ by adding an ethanolic solution of a stoichiometric amount of CuCl_2_ to the chloride salt of the cation dissolved in a minimum volume of 2-propanol. The solution is evaporated and the remaining complex is washed with diisopropyl ether [37]; (3) For a third procedure CuCl_2_ is added to an ethanolic solution of the chloride salt of the appropriate cation. The reaction mixture is heated under reflux for one hour and the product is precipitated by evaporating the solvent [38].

### 3.3. Bis(hexadecyltrimethylammonium) Tetrachloridocuprate(II), (C_16_H_33_Me_3_N)_2_[CuCl_4_] (**1**)

According to the second synthesis approach, for complex **1** 2.0 mmol (0.64 g) of C_16_H_33_Me_3_NCl were dissolved in 1.5 mL of 2-propanol and 1.0 mmol (0.14 g) of copper(II) chloride were dissolved in 0.3 mL of ethanol. Subsequently, both solutions were combined and cooled. An orange-red powder was obtained and separated by filtration.

Melting point: 85–86 °C. Yield: 0.74 g (95%). Elemental analysis calculated for C_38_H_84_N_2_CuCl_4_ (774.45): C 58.93, H 10.93, N 3.62 (%); found: C 58.17, H 11.27, N 3.61 (%). IR (KBr, cm^−1^): 2953 m, 2919 s, 2850 s, 1468 m, 963 m, 911 w, 721 w.

### 3.4. Bis(tetradecyltrimethylammonium) Tetrachloridocuprate(II), (C_14_H_29_Me_3_N)_2_[CuCl_4_] (**2**)

The complex **2** was prepared following the second procedure briefly mentioned above. 1.0 mmol (0.14 g) of copper(II) chloride, previously dissolved in 0.3 mL of ethanol, were added to 2.0 mmol (0.58 g) of C_14_H_29_Me_3_NCl dissolved in 1.5 mL of 2-propanol. The complex precipitated as orange powder.

Melting point: 83–85 °C. Yield: 0.61 g (85%). Elemental analysis calculated for C_34_H_76_N_2_CuCl_4_ (718.35): C 56.84, H 10.66, N 3.90 (%); found: C 58.11, H 11.03, N 4.15 (%). IR (KBr, cm^−1^): 2952 m, 2920 s, 2850 s, 1468 m, 966 w, 911 w, 722 w.

### 3.5. Bis(tetrabutylammonium) Tetrachloridocuprate(II), (Bu_4_N)_2_[CuCl_4_] (**3**)

This complex was synthesized via the third procedure using the following quantities: 0.5 mmol (0.07 g) of CuCl_2_ were added to 1.0 mmol (0.28 g) of Bu_4_NCl dissolved in 6 mL of ethanol and heated under reflux for one hour. Afterwards the alcoholic solution was evaporated until a dark red powder precipitated.

Melting point: 94–98 °C. Yield: 0.20 g (58%). Elemental analysis calculated for C_32_H_72_N_2_CuCl_4_ (690.29): C 55.68, H 10.51, N 4.06 (%); found: C 53.62, H 11.35, N 3.93 (%). IR (KBr, cm^−1^): 2962 s, 2873 ms, 1485 m, 1462 w, 1382 m, 881 m, 802 w, 739 mw.

### 3.6. Bis(benzyltriethylammonium) Tetrachloridocuprate(II), (BzlEt_3_N)_2_[CuCl_4_] (**4**)

Following the first synthesis, for complex **4** 1.0 mmol (0.23 g) of BzlEt_3_NCl were dissolved in 5 mL of 2-propanol and mixed with 0.5 mmol (0.09 g) of copper(II) chloride dihydrate, previously dissolved in 0.6 mL of hydrochloric acid. The mixture was heated for 30 min, after that the solution was evaporated. A yellow powder was obtained and washed with diisopropyl ether.

Melting point: 107–109 °C. Yield: 0.20 g (69%). Elemental analysis calculated for C_26_H_44_N_2_CuCl_4_ (590.00): C 52.93, H 7.52, N 4.75 (%), found: C 52.61, H 7.01, N 4.74 (%). IR (KBr, cm^−1^): 3059 w, 2985 m, 2944 w, 1582 w, 1479 s, 1453 w, 1390 ms, 1210 m, 1153 s, 1007 s, 900 m, 792 s, 752 s, 708 s, 603 w, 537 w, 462 w.

The UV/Vis spectra of all four compounds in solution (CH_3_CN, λ_max_/nm) are nearly identical with three characteristic absorptions: 256, 311, 462 nm.

## 4. Conclusions

A series of four potential high temperature ionic liquids on the basis of tetrachloridocuprate(II) anions with different sterically demanding cations has been studied by EPR spectroscopy as solid samples. These large cations are necessary for a reasonable separation of the paramagnetic Cu(II) centers. The spectra are all of axial symmetry. At lower temperatures (150 K) they show no resolved hyperfine pattern, but allow the determination of the *g*-tensor main values *g_║_* and *g*_┴_. From these values it is also possible to calculate the averaged *g*-values *g*_av_ which are comparable to the isotropic *g*-values of liquid systems. According to possible saturation effects, the signal in the EPR spectra decreases with increasing temperature and totally disappears at temperatures higher than about 350 K. This behavior is completely reversible and proves the thermal stability of these compounds at least up to 390 K.

From the very close UV/Vis absorption bands, one can conclude that all complexes in this series exhibit related coordination geometries of the [CuCl_4_]^2−^ unit. The compounds **1**–**4** do not show any thermochromic behavior up to 390 K. Even though the solid substances show slightly different colors in solid state, the UV/Vis spectra are nearly identical, indicating structural changes of the tetrachloridocuprate moieties between solid state and solution. The compounds have a promising potential e.g., as high temperature ionic liquids, as precursors for the formation of copper chloride particles or as catalytic paramagnetic ionic liquids.

## Figures and Tables

**Table 1:** Experimental EPR parameters (*g_║_*- and *g*_┴_-values) measured at 150 K.

Compound	*g*_║_	*g*_┴_	*g*_av_
(C_16_H_33_Me_3_N)_2_[CuCl_4_] (**1**)	2.365 ± 0.005	2.145 ± 0.005	2.218 ± 0.005
(C_14_H_29_Me_3_N)_2_[CuCl_4_] (**2**)	2.366 ± 0.005	2.141 ± 0.005	2.216 ± 0.005
(Bu_4_N)_2_[CuCl_4_] (**3**)	2.394 ± 0.005	2.081 ± 0.005	2.185 ± 0.005
(BzlEt_3_N)_2_[CuCl_4_] (**4**)	2.424 ± 0.005	2.089 ± 0.005	2.200 ± 0.005
(BuPy)_2_[Cu/ZnCl_4_] [33]	2.300 ± 0.005	2.070 ± 0.005	2.147 ± 0.005

## References

[1] Taubert A. (2009). Heavy elements in ionic liquids. Top. Curr. Chem.

[2] Taubert A. (2004). CuCl nanoplatelets from an ionic liquid-crystal precursor. Angew. Chem. Int. Ed.

[3] Saito G, Ohno H. (2011). Electric Conductivity and Magnetic Ionic Liquids. Electrochemical Aspects of Ionic Liquids.

[4] Kalb R (2008). Use of Magnetic, Ionic Liquids as an Extraction Agent. International Application No.: PCT/EP2008/067731.

[5] Dengler J.E., Doroodian A., Rieger B. (2011). Protic metal-containing ionic liquids as catalysts: Cooperative effects between anion and cation. J. Organomet. Chem.

[6] Bica K., Gaertner P. (2008). Metal-containing ionic liquids as efficient catalysts for hydroxymethylation in water. Eur. J. Org. Chem.

[7] Bica K., Gaertner P. (2006). An iron-containing ionic liquid as recyclable catalyst for aryl grignard cross-coupling of aryl halides. Org. Lett.

[8] Lin I.J.B., Vasam C.S. (2005). Metal-containing ionic liquids and ionic liquid crystals based on imidazolium moiety. J. Organomet. Chem.

[9] Bowlas C.J., Bruce D.W., Seddon K.R. (1996). Liquid-crystalline ionic liquids. Chem. Commun.

[10] Neve F., Francescangeli O., Crispini A., Charmant J. (2001). A_2_[MX_4_] copper(II) pyridinium salts. From ionic liquids to layered solids to liquid crystals. Chem. Mater.

[11] Neve F., Francescangeli O., Crispini A. (2002). Crystal architecture and mesophase structure of long-chain *N*-alkylpyridinium tetrachlorometallates. Inorg. Chim. Acta.

[12] Neve F., Crispini A. (2001). C-H···Br-M interactions at work: Tetrabromometalates of the bolaamphiphilic *N*,*N′*-dodecamethylenedipyridinium cation. Cryst. Growth Des.

[13] Neve F., Crispini A. (2001). C-H···Br-M interactions at work: Tetrabromometalates of the bolaamphiphilic *N*,*N′*-dodecamethylenedipyridinium cation (correction). Cryst. Growth Des.

[14] Taubert A., Palivan C., Casse O., Gozzo F., Schmitt B. (2007). Ionic liquid-crystal precursors (ILCPs) for CuCl platelets: The origin of the exothermic peak in the DSC curves. J. Phys. Chem. C.

[15] Taubert A., Steiner P., Mantion A. (2005). Ionic liquid crystal precursors for inorganic particles: Phase diagram and thermal properties of a CuCl nanoplatelet precursor. J. Phys. Chem. B.

[16] Willett R.D., Haugen J.A., Lebsack J., Morrey J. (1974). Thermochromism in copper(II) chlorides. Coordination geometry. Inorg. Chem.

[17] Zhuravlev O.E., Verolainen N.V., Voronchikhina L.I. (2011). Thermal stability of 1,3-disubstituted imidazolium tetrachloroferrates, magnetic ionic liquids. Russ. J. Appl. Chem.

[18] Branco A., Branco L.C., Pina F. (2011). Electrochromic and magnetic ionic liquids. Chem. Commun.

[19] Neve F., Crispini A., Armentano S. (1998). Synthesis, structure, and thermotropic mesomorphism of layered *N*-alkylpyridinium tetrahalopalladate(II) salts. Chem. Mater.

[20] Neve F., Francescangeli O. (2005). Layered ω-substituted alkylpyridinium salts with inorganic anions: Effects of H-bonding patterns on the layer thickness. Cryst. Growth Des.

[21] Neve F., Crispini A. (2007). Competitive interactions in carboxy-functionalized pyridinium salts: Crossover from O-H···O to O-H···X-M contacts. Cryst. Eng. Comm.

[22] Neve F., Crispini A. (2003). *N*,*N*′-Dodecamethylene-bis(pyridinium) goes lamellar. Role of C-H···I, C-H···M, and I···I interactions in the crystal structure of its hexaiododipalladate(II) derivative. Cryst. Eng. Comm.

[23] Neve F., Crispini A., Francescangeli O. (2000). Structural studies on layered alkylpyridinium iodopalladate networks. Inorg. Chem..

[24] Binnemans K. (2005). Ionic liquid crystals. Chem. Rev.

[25] Goossens K., Lava K., Nockemann P., van Hecke K., van Meervelt L., Driesen K., Görller-Walrand C., Binnemans K., Cardinaels C. (2009). Pyrrolidinium ionic liquid crystals. Chem. Eur. J.

[26] Martinez Casado F.J., Ramos Riesco M., da Silva I., Labrador A., Redondo M.I., Garcia Perez M.V., Lopez-Andres S., Rodriguez Cheda J.A. (2010). Thermal and structural study of the crystal phases and mesophases in the lithium and thallium(I) propanoates and pentanoates binary systems: Formation of mixed salts and stabilization of the ionic liquid crystal phase. J. Phys. Chem. B.

[27] Suisse J.-M., Douce L., Bellemin-Laponnaz S., Maisse-Francois A., Welter R., Miyake Y., Shimizu Y (2007). Liquid crystal imidazolium salts: Towards materials for catalysis and molecular electronics. Eur. J. Inorg. Chem.

[28] Dobbs W., Suisse J.-M., Douce L., Welter R. (2006). Electrodeposition of silver particles and gold nanoparticles from ionic liquid-crystal precursors. Angew. Chem. Int. Ed.

[29] Lee C.K., Peng H.H., Lin I.J.B. (2004). Liquid crystals of *N*,*N′*-dialkylimidazolium salts comprising palladium(II) and copper(II) ions. Chem. Mater.

[30] Herber R.H., Nowik I., Kostner M.E., Kahlenberg V., Kreutz C., Laus G., Schottenberger H. (2011). Mössbauer spectroscopy and x-ray diffraction study of ^57^Fe-labeled tetrachloroferrate(III)-based magnetic ionic liquids. Int. J. Mol. Sci.

[31] De Pedro I., Rojas D.P., Albo J., Luis P., Irabien A., Blanco J.A., Fernandez J.R. (2010). Long-range magnetic ordering in magnetic ionic liquid: Emim[FeCl_4_]. J. Phys. Condens. Matter.

[32] Sasaki T., Zhong C., Tada M., Iwasawa Y. (2005). Immobilized metal ion-containing ionic liquids: Preparation, structure and catalytic performance in Kharasch addition reaction. Chem. Commun.

[33] Thiel K., Klamroth T., Strauch P., Taubert A. (2011). On the interaction of ascorbic acid and the tetrachlorocuprate ion [CuCl_4_]^2−^ in CuCl nanoplatelet formation from an ionic liquid precursor (ILP). Phys. Chem. Chem. Phys.

[34] Farra R., Thiel K., Winter A., Klamroth T., Pöppl A., Kelling A., Schilde U., Taubert A., Strauch P. (2011). Tetrahalidocuprates(II)—Structure and EPR spectroscopy. Part 1: Tetrabromidocuprates(II). New J. Chem.

[35] Smith D.W. (1976). Chlorocuprates(II). Coord. Chem. Rev.

[36] Liu H., Wang X., Hu W., Guo L, Ouyang S. (2004). Preparation and characterization of (C_4_H_9_NH_3_)_2_CuX_4_ (X-Cl, Br). Chem. J. Internet.

[37] Choi S., Larrabee J.A. (1989). Thermochromic tetrachlorocuprate(II). J. Chem. Educ.

[38] Xiao Z.-L., Chen H.-Z., Shi M.-M., Wu G., Zhou R.-J., Yang Z.-S., Wang M., Tang B.-Z. (2005). Preparation and characterization of organic-inorganic hybrid perovskite (C_4_H_9_NH_3_)_2_CuCl_4_. Mater. Sci. Eng. B.

